# Use of antibiotics in rural and urban regions in the Netherlands: an observational drug utilization study

**DOI:** 10.1186/1471-2458-14-677

**Published:** 2014-07-03

**Authors:** Josta de Jong, Jens HJ Bos, Tjalling W de Vries, Lolkje TW de Jong-van den Berg

**Affiliations:** 1 Department of Pharmacoepidemiology and Pharmacoeconomics, University of Groningen, A. Deusinglaan 1, 9713AV Groningen, the Netherlands; 2 Medical Centre Leeuwarden, Henri Dunantweg 2, 8934 AD Leeuwarden, the Netherlands

**Keywords:** Antibiotics, Cattle, Bacterial resistance, Humans, Rural, Urban

## Abstract

**Background:**

Large livestock farms might increase the infection risk for the nearby human population because of an increased risk for disease outbreaks and because antibiotic-resistant bacteria are more likely to be present. We hypothesized that populations residing in rural areas have more contact with cattle compared with populations in urban areas, and will use more antibiotics or more frequently require a new course of antibiotics.

**Methods:**

Using data from the prescription database IADB.nl, we compared antibiotic use by patients living in rural areas to the use by patients living in urban areas. We also followed cohorts of antibiotic users and determined the patients who required a second antibiotic within 14 days after beginning the first antibiotic.

**Results:**

The yearly prevalence of antibiotic use was greater in rural areas compared with urban areas (2009: 23.6% versus 20.2% (p < 0.001), especially in the younger age groups. More adult patients residing in rural areas required a second course of antibiotic treatment within 14 days after starting the first treatment.

**Conclusion:**

Individuals use more antibiotics, and adults more frequently require a second antibiotic prescription within 14 days, in rural areas compared with urban areas. Although the differences were small and the risks for the general rural population were not high, this difference should be investigated further.

## Key points

• Individuals residing in rural areas in the Netherlands used more antibiotics, especially young people < 45 years of age.

• Adults in rural areas were more likely to require a second course of antibiotics within 14 days after starting the first course.

• The findings of this study might result from greater exposure to resistant bacteria that originate from cattle farms.

## Background

Concerns about the health of individuals who work at or live near livestock farms have been increasing in the Netherlands. Over the last 20 years, the number of farms have decreased while the numbers of animals have increased. This increase has been especially significant for animals (e.g., poultry, pigs), which are kept in large numbers in concentrated growout facilities [1]. Concentrations of large numbers of animals could contribute to an increased risk of outbreaks of diseases, such as avian and swine influenzas, foot-and-mouth-disease, and Q fever. Increased within-population transmission of bacteria, development of resistance via the increased use of veterinary antibiotics, and the concentration of fine dust near livestock farms are consequences of this intensive animal production.

In 2009, the nearby human population was affected by an outbreak of Q fever (*Coxiella burnettii*) in goats in the southern Netherlands [2]. The discovery of a livestock-associated multi-drug resistant *Staphylococcus aureus* (MRSA) [3,4] resulted in revision of the guidelines for individuals who work with cattle and become hospitalized [5]. Highly resistant *Escherichia coli* strains from dairy farms have caused serious infections in humans in Belgium and the United States [6,7].

In addition to these specific outbreaks, there may be general effects on the health of populations living in areas near livestock farms. There is little information about whether, compared with people in urban areas and others who are not typically in close contact with cattle, infections are more common, antibiotic use is greater, and antibiotics are more likely to be ineffective in populations that reside in rural areas.

Using a pharmacy prescription database, we investigated the differences in antibiotic use between urban and rural areas. We used the need for a new antibiotic prescription within 14 days after starting a course of treatment as a signal for therapeutic failure.

## Methods

### The IADB.nl database

Information on drug use was obtained from the IADB.nl database, which contains pharmacy-dispensing data from community pharmacies in the Netherlands. Dutch patients usually register at a single community pharmacy, so a single pharmacy provided an almost complete listing of each subject’s prescribed drugs [8]. The pharmacy data included information on the name of the drug dispensed, the Anatomical Therapeutic Chemical (ATC) classification, a prescription date, the number of days the drug was prescribed, and the number of defined daily doses. The characteristics of these data were based on World Health Organization definitions [9]. Over-the-counter drugs and in-hospital prescriptions were not included. The database contains the prescriptions of a population of 500,000 individuals.

The data in the IADB.nl database were strictly anonymous. No identification of individuals was possible. According to Dutch regulations, ethical approval was not required for the study [8].

### Degree of urbanization

To perform this study, we required additional information on the environments in which the subjects resided. We used degree of urbanization (DU) information maintained by Statistics Netherlands (Centraal Bureau voor de Statistiek, Den Haag/Heerlen, the Netherlands) [10]. In the Statistics Netherlands database, the DU value varied from 1 (extremely urbanized; 2,500 or more addresses per square kilometer) to 5 (not urbanized; fewer than 500 addresses per square kilometer). The DU value was available for every neighborhood within a community.

Every prescription in the IADB.nl database was linked to the first four numbers of each subject’s postal code (Postal Code-4). We selected all Postal Code-4 areas in which ≥60% of the population was included in the IADB.nl database.

The DU was linked to neighborhoods in the Statistics Netherlands database, not to postal code areas. There was only partial correspondence, and in most cases differences and overlaps were present. To resolve this problem, we determined the neighborhood with the lowest DU value (i.e., the most urbanized) in each Postal Code-4 area. The Postal Code-4 area was then assigned this DU value. To maintain anonymity of the data, the postal codes were deleted from the data set after the DU values were assigned.

To find an optimal effect, we selected the Postal Code-4 areas with a DU of 1 (n = 24) (‘urban’) and with a DU of 5 (n = 28) (‘rural’). The rural areas were identified as agricultural areas using the software application Google Earth (©2011 Google Inc., Mountain View, CA, USA). We selected all antibiotic drug prescriptions that were dispensed in these areas (ATC-code starting with J01) between 1998 and 2009.

### Analysis of the number of prescriptions and types of antibiotics

The number of prescriptions per person residing in both the rural and the urban populations was determined for each year (1998 to 2009). We specifically examined the prescriptions for 1999, 2004, and 2009, and calculated the prevalence of the population using at least one course of antibiotics per year, stratified by age and sex. For these 3 years, we also calculated the average number of prescriptions per antibiotic user. We also determined the proportions of the prescriptions of the different types of antibiotics for the rural and urban populations.

### Therapeutic failure

Therapeutic failure was investigated by determining whether a second course of antibiotics was prescribed within 14 days of the start of a new course of antibiotics. A course of antibiotics was defined as new if the patient was not prescribed antibiotics during the preceding 30 days. To determine therapeutic failure, two cohorts of patients (one rural and one urban) who started an antibiotic course between 1 January 1999 and 31 December 2009 were selected. We compared the rural cohort with the urban cohort in an age-stratified analysis, and estimated the relative risks (RR) of needing a new prescription within 14 days after beginning the new treatment.

### Statistical analysis

The Chi-square test was used to compare percentages. The Mann-Whitney *U* test was used to compare the numbers of prescriptions per person. Relative risks (RRs) with 95% confidence intervals (CI) were used to compare therapeutic failure. Microsoft® Office Excel 2010 (Microsoft® Corp., Redmond, WA, USA), SPSS 16.0 for Windows (IBM Corp., Armonk, NY, USA), and R version 2.13.0 (Free Software, Free Software Foundation, Boston, MA, USA) software were used for the data analysis. A p-value < 0.05 was considered to be statistically significant.

## Results

**Figure 1:**
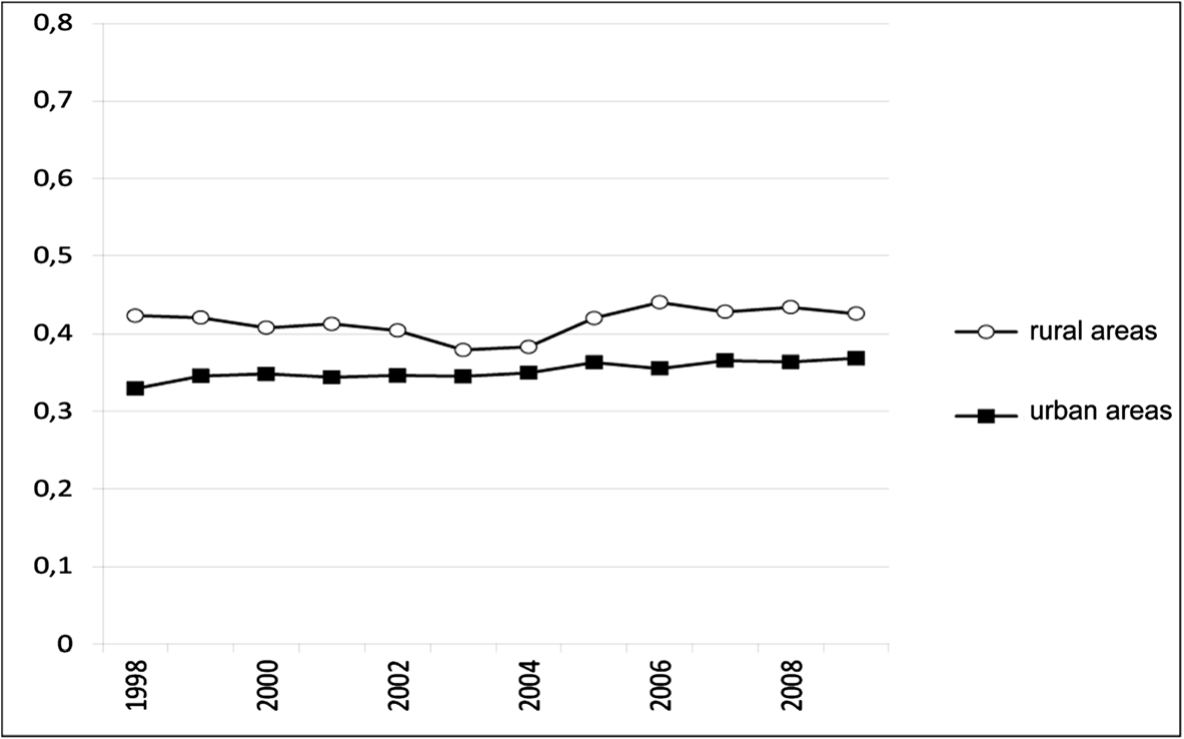
Number of antibiotics prescriptions per person, per year, for the rural (degree of urbanization (DU) = 5) and the urban (DU = 1) populations.

A total of 141,866 prescriptions for antibiotics for Postal Code-4 areas with a DU value of 5 (‘rural’) were selected from the database. A total of 569,946 antibiotics prescriptions for Postal Code-4 areas with a DU value of 1 (‘urban’) were selected. The results for the 2009 data for population characteristics are presented in Table 1. The rural areas had a much higher percentage of children, and adults between 46 and 70, compared with the urban areas. The 20–45-year age group was the most highly represented age group in the urban areas. There were more males than females in the rural areas.Figure 1 presents the results for the average number of prescriptions per inhabitant for the years 1998 to 2009. Antibiotics were prescribed more frequently in rural areas for each year.

**Table 1:** Characteristics of the prescriptions and of the study population, 2009

	**Rural (DU = 5)**	**Urban (DU = 1)**	p*
**Number of prescriptions**	141 866	569 946	
	(1998-2009)	(1998-2009)	
**Number of patients in the population (2009)**	37 896	140 726	
**Age distribution of patients (2009)**			
**0-19 years**	23.5% (8,918)	15.7% (22,132)	<0.001
**20-45 years**	30.3% (11,500)	51.9% (73,169)	<0.001
**46-70 years**	36.9% (13,997)	23.5% (33,271)	<0.001
**71 years or older**	9.2% (3,481)	8.8% (12,414)	0.028
**Males in the population (2009)**	50,2% (19,033)	47,6% (67,106)	<0.001

^*^X^2^ test.

Table 2 presents the results for the prevalence of antibiotics users in the study population. In the two younger age groups, the prevalence was significantly higher in the rural areas in 1999, 2004, and 2009. There was no difference in prevalence for the 46–70-year age group in 1999 or 2009. In 2004 and 2009, the user prevalence for the ≥71 age group was higher in the urban areas. For the entire group, and for male and female subjects separately, the prevalence of antibiotics users was higher in the rural areas.

**Table 2:** Prevalence (%) of persons using antibiotics, stratified by 3 separate years and by age and sex

**Year/age group**	**1999**			**2004**			**2009**		
	**Rural**	**Urban**	p*	**Rural**	**Urban**	**p**	**Rural**	**Urban**	**p**
**Total**	**24.8**	**20.2**	**<0.001**	**22.4**	**19.8**	**<0.001**	**23.6**	**20.2**	**<0.001**
**0-19 years**	**23.9**	**18.6**	**<0.001**	**20.6**	**18.1**	**<0.001**	**19.3**	**17.4**	**<0.001**
**20-45 years**	**23.0**	**15.9**	**<0.001**	**20.3**	**15.8**	**<0.001**	**22.6**	**16.1**	**<0.001**
**46-70 years**	25.3	24.8	0.413	**23.2**	**22.7**	**<0.001**	23.5	23.4	0.9822
**71 and older**	**38.1**	**33.7**	**<0.001**	*34.0*	*36.5*	*0.015*	*36.6*	*41.9*	*<0.001*
**male**	**21.2**	**16.1**	**<0.001**	**19.1**	**15.3**	**<0.001**	**19.7**	**15.7**	**<0.001**
**female**	**28.5**	**23.7**	**<0.001**	**25.5**	**23.5**	**<0.001**	**27.6**	**24.2**	**<0.001**

^*^X^2^ test. **Bold**: significant difference, higher in rural area; *Italics*: significant difference higher in urban area.

The results for the number of prescriptions per antibiotics user are presented in Table 3. There was no difference between the two older groups or (in 2004), a slightly greater number of antibiotics were prescribed in the urban areas. The number of prescriptions per antibiotics user was significantly higher in rural areas in the age group ≤45 years. In 2009, significantly greater numbers of antibiotics were prescribed per user among the entire group, and among males, in rural areas. A similar result occurred in females in 1999.

**Table 3:** Average number of prescriptions per year, per user of antibiotics, stratified by 3 separate years and by age and sex

**Year/age group**	**1999**			**2004**			**2009**		
	**Rural**	**Urban**	p^*1^	**Rural**	**Urban**	**p**	**Rural**	**Urban**	**p**
**Total**	1.70	1.71	0.400	1.72	1.76	0.525	**1.80**	**1.82**	**<0.001**
**0-19 years**	**1.64**	**1.47**	**<0.001**	**1.58**	**1.50**	**0.002**	**1.55**	**1.47**	**<0.001**
**20-45 years**	**1.55**	**1.49**	**0.003**	**1.56**	**1.50**	**0.001**	**1.57**	**1.47**	**<0.001**
**46-70 years**	1.72	1.80	0.129	*1.75*	*1.83*	*0.008*	1.79	1.81	0.802
**71 and older**	2.28	2.22	0.606	*2.29*	*2.44*	*0.047*	2.66	2.84	0.533
**male**	1.60	1.63	0.332	1.62	1.66	0.329	**1.65**	**1.63**	**0.019**
**female**	**1.77**	**1.76**	**0.022**	1.79	1.83	0.582	1.91^*2^	*1.91*	*<0.001*

^*1^Mann-Whitney *U* test. **Bold**: significant difference, higher in rural area; *Italics*: significant difference higher in urban area.

^*2^Rural: 1.912, urban: 1.914.

The cephalosporins and the fluoroquinolones are mostly distinguished as ‘reserved’ antibiotics to prevent antibiotic resistance. These two types of antibiotics were prescribed more often in urban areas (Table 4). Compared with rural areas, sulfonamide/trimethoprim was prescribed more often in urban areas. In rural areas, the tetracyclines, penicillins, and macrolids were prescribed more often than other antibiotics.

**Table 4:** Proportions of the different antibiotic groups (number of prescriptions)

	**Rural (n = 183 947)**	**%**	**Urban (n = 626 584)**	**%**	P*
**J01A (tetracyclins)**	42 604	23.16	13 4594	21.48	<0.001
**J01B (amfenicoles)**	3	0.002	9	0.001	0.877
**J01C (penicillins)**	71 644	38.95	20 1984	32.24	<0.001
**J01D(cephalosporins)**	1012	0.55	4520	0.72	<0.001
**J01E (sulfonamide/trimethoprim)**	20 025	10.89	88 906	14.19	<0.001
**J01F (macrolids)**	22 025	11.97	67 981	10.85	<0.001
**J01G (aminoglycosids)**	323	0.18	510	0.08	<0.001
**J01M (fluorochinolons)**	10 163	5.52	53 375	8.52	<0.001
**J01X (miscellaneous)**	16 148	8.79	74 705	11.92	<0.001

^*^X^2^ test.

The estimated RRs for being prescribed a second course of antibiotics within 14 days of beginning a new antibiotic are presented in Table 5. The RRs did not significantly differ from 1 for the youngest age group. In the older age groups, the RRs were significantly greater than 1, but the differences were small.

**Table 5:** Number of patients in the cohort of subjects who were prescribed a second course of antibiotics within 14 days after the initial course

**1999-2005**					
	**Rural**		**Urban**		
**Age**	**Nr new antibiotic**	**Total nr patients**	**Nr new antibiotic**	**Total nr patients**	**Relative risk (Confidence intervals)**
	**In 14 days**		**In 14 days**		
0-19	1902	23 955	3733	49 128	1.04 (0.99-1.10)
**20-40**	**2409**	**25 152**	**11 426**	**127 056**	**1.06 (1.02-1.11)**
**41-60**	**3863**	**33 452**	**8591**	**83 736**	**1.12 (1.09-1.17)**
**60-80**	**3435**	**24 994**	**11 014**	**87 526**	**1.09 (1.05-1.13)**
**Total**	**11609**	**107553**	**34764**	**347446**	**1.08 (1.06-1.10)**

Relative risks of patients in rural areas compared with those in urban areas.

**Bold**: significant difference.

## Discussion

We found that the use of antibiotics per year was higher in rural areas compared with urban areas (Figure 1). This difference mainly applied to individuals <46 years of age. No significant differences were found in the older age groups. In fact, in urban areas the antibiotic use was even greater (Tables 2 and 3). In rural areas, it was more customary to begin another course of antibiotics within 14 days after starting the initial antibiotic (Table 5), but this difference did not apply to the youngest group. There was also a difference in the type of antibiotic drugs prescribed. ‘Reserved’ antibiotics were prescribed more often in urban areas. This result did not support our hypothesis that these types of drugs would be more likely to be prescribed in rural areas (Table 4).

The empirical probability of medication use, especially antibiotics, is also associated with socioeconomic status [11,12]. In many countries, the socioeconomic status in rural areas is lower compared with urban areas. In the Netherlands, populations with lower socioeconomic status tend to be present in the most urbanized areas [13]. Therefore, socioeconomic differences cannot explain the higher number of antibiotics prescriptions in rural areas. The age distribution (Table 1) results indicate that there were more individuals aged 20 through 45 years living in urban areas. This result could be attributed to the fact that the largest city in the IADB-population is Groningen, which is a university town. A young population in the urban area could be a factor that contributed to the lower use of antibiotics, but the results in Tables 2 and 3 indicate that the difference between the two areas mostly occurs in the younger groups.

The use of cephalosporins and fluoroquinolones was more frequent in urban areas, which was not the result we expected. The close proximity of hospitals and the availability of specialized care could have contributed to this result.

No differences were found in children for the RR of needing another antibiotic, but the RR significantly exceeded 1 in the older age groups. Therapeutic failure of antibiotics seems to occur more often in older individuals living in rural areas, compared with those living in urban areas. The RR values were very close to 1, which implies that the measured effect was small. The effect might have been diluted by rural patients who did not have regular contact with cattle.

No other studies have been published for populations from the Netherlands that compare the use of antibiotics in urban and rural areas. Because urban and rural areas are characteristically very dissimilar in different countries, the results of studies performed in other countries are difficult to compare with the results of our study.

Two studies that investigated health symptoms associated with rural areas were performed in the Netherlands. Investigators that examined a population in the southern Netherlands compared the health of individuals residing near intensive livestock farms with that of individuals in other regions [14]. They reported that there were no relevant differences. The results of this previous study suggest that compared with the reference group, fewer people near intensive livestock farms suffer from asthma, but respiratory infections in humans with asthma are more frequent. Pneumonia occurs more often near farms, which the authors related to the Q fever epidemic that occurred in that area. Children with eczema were also more likely to reside near farms. In the second study, which investigated rural-urban health differences, rural areas had a higher prevalence of infections. Working in close contact with animals was proposed as a cause for the difference [14]. Similar to our results, both studies found only small differences between groups. The health risk for becoming infected that results from residing near livestock farms is probably not large in the general population in rural areas. The effect we found could have been diluted, because we had no specific information about individual contact with animals. These details should be investigated further in groups where more information is available about contact with animals and distance of a residence from farms. It is possible that for certain groups (e.g., individuals with asthma or the elderly) the risk could increase over time, as resistance against antibiotics disseminates in susceptible populations. It is important to note that in our study population children and younger people used more antibiotics, but the older age groups required a second course of antibiotic treatment more often.

Two advantages of our study were that we used objective pharmacy data and large population sizes. One study limitation was that some specific population data was missing. The database consisted of pharmacy prescription data, but did not include information on indications. The study was also limited by its assumption that inhabitants of rural areas were more likely to reside in closer proximity to cattle. We assumed that because most farmers reside on their farms, the chance that a patient worked with animals was greater compared with urban residents.

## Conclusion

Compared with young people residing in urban areas, young people that resided in rural areas were more likely to use antibiotic drugs. Adults in rural areas more frequently required a second prescription of antibiotics within 14 days after beginning the first prescription. This result suggests that therapeutic failure due to antibiotic resistance may be a factor in this group. The differences between groups were quite small, probably because the effects were diluted. The risk for the general rural population is probably not significant. However, the factors that may have contributed to these results should be investigated in greater detail.

### Ethical approval

The data in the IADB.nl database are strictly anonymous. No derivation to any recognizable person is possible. Ethical approval was not required, according to Dutch regulations.

## Competing interests

The authors declare that they have no competing interests.

## Authors’ contributions

JJ performed the study and wrote the manuscript. JB, an IT-specialist and programmer, developed the databases and assisted in selection and analysis of the data. TV participated in the design of the study, and in the analysis and preparation of the manuscript. LJB supervised and provided advice during the performance of this study. All authors read and approved the final manuscript.

## Pre-publication history

The pre-publication history for this paper can be accessed here:

http://www.biomedcentral.com/1471-2458/14/677/prepub
